# Supplementary material for: Research on the generation of food safety network public opinion—Taking the Chinese Mouse Head Duck Neck Incident as an example
Source: PLoS One. 2025 Jul 28;20(7):e0324876. doi: 10.1371/journal.pone.0324876 (PMC12303279; doi:10.1371/journal.pone.0324876)
Supplement: Attachment — Submitted filename: Response to Reviewers.docx [file pone.0324876.s001.docx]

We are deeply grateful for your thorough and insightful review of our article. Following your valuable suggestions, we have made extensive revisions to our initial draft. The detailed corrections and improvements are outlined below.

**I have made the following changes to the suggestions made by the first reviewer:**

With regard to the suggested changes to the literature review section, we have revised the literature review section by reorganizing the content to review previous studies in a more analytical way, rather than listing the studies in chronological order. The literature review is now more critical and comprehensive and clearly identifies gaps in current knowledge as well as the unique contributions of this study.

Regarding the section on methodology, it has been appropriately streamlined in the revised text, while regarding the identification of the number of topics, the reasons for choosing perplexity as an assessment metric for topic modeling and the advantages of perplexity over other options have been clearly stated in the paper.

Regarding the section on deepening the discussion of the analysis, in response to the reviewers’ suggestions, a detailed explanation of the transition has been provided before proceeding with the coding of the grounded theory to ensure the depth and coherence of the analysis.

In the section on strengthening practical implications, specific insights have been added based on the analysis of the paper and the summary and recommendations section at the end of the article has been revised to enhance the practical value and flow of the paper.

**I have made the following changes to the suggestions made by the second reviewer:**

The writing of the literature review has been optimized based on the reviewers’ suggestions by using clearer, simpler sentences and using technical terms only when necessary to improve readability and comprehension.

**I have made the following changes to the suggestions made by the third reviewer:**

On the issue of social media posts, a discussion of the ethics of using social media posts has been added to the data collection section, and transparency has been ensured on this.

Regarding the issue of deleting emojis, this has been explained in the preprocessing section of the revised text.

Regarding the merging of synonyms in the word cloud section, the merging was done manually as only the top 100 words in terms of frequency were selected for this paper. The definition of co-occurring phrases in this paper is phrases that co-occur in a single comment, and as for the semantic relationship network Figure in the paper, it has also been drawn based on the frequency of co-occurrence of words, and has been based on the reviewer’s suggestion of using a more active tense and the first person in the paper to describe the different aspects of the study in order to show how the study was conducted in a clearer way.

The range of the number of topics chosen in the paper for the confusion figure is 0-15, 15 is not a fixed value, just make sure that the optimum number of topics is between the ranges but it should not be chosen to be too large or too small; also the paper has not chosen any other method to determine the number of topics for the reasons explained in the perplexity section of the revised paper.

The advantages and disadvantages of the methods used in this paper have been discussed in the summary section at the end of the revised text.

The explanation from spatial coding to axial coding has been reflected in the revised text: the initial categories are generalized and enhanced by combining the coding results of previous researchers. The similarities and differences between the theoretical memos in traditional grounded theory and the methods we used in our study have been discussed in detail in the model building section of the revised text.

Regarding the reviewer’s suggestion to use the COREQ framework to evaluate this paper: first of all, we are very grateful to the reviewer for this valuable suggestion, and since it is not easy to list all the 32 entries in this paper, this paper only lists some of the core entries such as the research questions, the data collection methods, and the data analyses, which have been interspersed with the various panels in the text.

**I have made the following changes to the suggestions made by the fourth reviewer:**

All figures and tables have been added in the revised text to support our analysis and conclusions.

In response to the comments made in the literature review section, we have added critical discussions and unique insights in the related work section as suggested and broken it down by research focus to improve readability and logic. We have also streamlined and optimized the lengthy content.

As suggested by the reviewers, we have simplified the theoretical descriptions on methodology and highlighted specific research steps and data processing methods

We thank the reviewers for their corrections and we have noted the inconsistency between the two. and have explained this in the revised text: the traditional grounded theory is not suitable for this paper, which is a combination of text mining and grounded theory.

The choice of tag words is determined by the topic words and the words with higher TF-IDF values. The six topics under the topic words in the text have been selected ten in Table 3 (of course, this is only part of the words under each topic), TF-IDF value of the highest ten words in the text is also listed in Figure 5, of course, the above is only a part of the tag words, the rest of the tag words are also selected from the topic words or words with high TF-IDF values. As the selection of tag words reaches 90, it is unrealistic to list all the tag words in the table of topic words and the figure of words with high TF-IDF value.

Regarding the reviewer’s suggestion about the lack of explicit logical reasoning and explanation in the coding process of grounded theory, it has been explained and modified in the revised text to ensure the consistency of the paper’s structure and logical integrity.

Thanks to the reviewers’ suggestions, we will check and adjust the reference formatting in strict accordance with the submission guidelines of the target journal to enhance the professional quality of the paper.

We will make linguistic touches and revisions to the paper as suggested to ensure that the clarity and readability of the text meets publication standards.

**I have made the following changes to the suggestions made by the fifth reviewer:**

Based on the reviewers’ suggestions, revisions have been made in the text: the limitations of traditional grounded theory have been emphasized, as well as the reasons for combining text mining and grounded theory in this paper, and in order to ensure the rigor of the coding, the process of coding has also been exchanged with some of the students and the coding results of previous researchers have been referred to.

Regarding the two references mentioned in the reviewers’ revisions, they have been cited and compared in the revised text, mentioning the strengths and weaknesses of these studies.

According to the reviewer’s suggestion, a categorization has been made in the literature review section for the different research methods on network public opinion and the unique features of the research methodology of this paper have been emphasized.

Changes have been made to the introduction of the LDA topic model in the revised text, and the entire research process has also been refined to improve reproducibility.

The categories identified in the model and the results of the analysis have been used as a basis for policy recommendations based on the reviewers’ revisions.

**I have made the following changes to the suggestions made by the sixth reviewer:**

Figure 1 presents mainly the technical roadmap of the paper, aiming to roughly show the research steps of this paper.

Thanks to the reviewer’s suggestion, the name of Figure 5 has been changed to Semantic Relationship Network Figure

As suggested by the reviewers, it has been specified how these subject terms under each topic support our explanations in the discussion.

Thanks to the reviewer’s suggestion, we have added a clear figure in 3.3 to explain grounded theory more clearly.

Following the reviewer’s suggestion, we will expand on the discussion in Section 4 by broadening the findings and implications to provide broader insights into public opinion formation on food safety.

We thank the reviewers for their suggestions for the literature review section, which has been revised in accordance with the revisions.

With regard to “network public opinion” and “online public opinion”, this has been harmonized in the revised text.
