# Supplementary material for: Research on the generation of food safety network public opinion—Taking the Chinese Mouse Head Duck Neck Incident as an example
Source: PLoS One. 2025 Jul 28;20(7):e0324876. doi: 10.1371/journal.pone.0324876 (PMC12303279; doi:10.1371/journal.pone.0324876)
Supplement: Attachment — Submitted filename: Response_to_Reviewers_auresp_2.docx [file pone.0324876.s002.docx]

We are deeply grateful to the reviewers for their thorough and insightful comments on our manuscript. Based on your invaluable suggestions, we have undertaken another round of revisions and refinements. The detailed corrections and improvements are outlined below.

**I have made the following changes to the suggestions made by the second reviewer:**

Thank you very much for your comments on the revision of the literature review section in the previous round of revisions, and thank you for recognizing our manuscript.

**I have made the following changes to the suggestions made by the third reviewer:**

Thank you very much for your valuable comments during the last round of revisions, indeed it is one of the highlights of this paper. At the same time, COREQ has a total of 32 entries, it is indeed unrealistic to use them completely in this paper, so only some core entries have been selected in this paper, thank you for your understanding.

**I have made the following changes to the suggestions made by the fifth reviewer:**

Thank you very much for the revisions you made during the last round of revisions, and thank you for recognizing our revision process and the revised manuscript.

**I have made the following changes to the suggestions made by the sixth reviewer:**

About how we rewrite the text.

Figure 1 would have presented the technology roadmap for this paper, but given the short length of this paper, it was decided to remove it.

Thanks to the reviewer’s suggestion, the name of Figure 5 has been changed to Semantic Relationship Network Figure

As suggested by the reviewer, we have shown how those subject terms under each theme support our explanations in the discussion (including in the context of text specifics).

Thanks to the reviewer’s suggestion, we have added a clear figure in Method section (Figure 2) to explain grounded theory more clearly.

Following the reviewer’s suggestion, we will expand on the discussion in Section 4 by broadening the findings and implications to provide broader insights into public opinion formation on food safety.

We thank the reviewers for their suggestions for the literature review section, which has been revised in accordance with the revisions.

With regard to “network public opinion” and “online public opinion”, this has been harmonized in the revised text.
